# Imitation in Angelman syndrome: the role of social engagement

**DOI:** 10.1038/s41598-020-72079-3

**Published:** 2020-10-02

**Authors:** Serena Micheletti, Giacomo Vivanti, Stefano Renzetti, Paola Martelli, Stefano Calza, “Imitation in Angelman” Study Group, Patrizia Accorsi, Anna Alessandrini, Nicole D’Adda, Micaela De Simone, Laura Ferrari, Valentina Foresti, Jessica Galli, Lucio Giordano, Elisa Scarano, Caterina Strobio, Elisa Fazzi

**Affiliations:** 1 Unit of Child Neurology and Psychiatry, ASST Spedali Civili of Brescia, Brescia, Italy; 2 AJ Drexel Autism Institute, Drexel University, Philadelphia, PA USA; 3 Unit of Biostatistics and Bioinformatics, Department of Molecular and Translational Medicine, University of Brescia, Brescia, Italy; 4 Department of Clinical and Experimental Sciences, University of Brescia, Brescia, Italy

**Keywords:** Neuroscience, Psychology

## Abstract

Individuals with Angelman syndrome (AS) are characterized by severe cognitive impairments alongside an enhanced drive for social engagement. As knowledge on imitation skills in this population is limited, we conducted the first controlled study of imitation in AS. We examined how 23 individuals with AS and 21 typically developing young children with similar mental age imitated novel actions in response to socially or non-socially engaging models, and in response to video-recorded versus live demonstrations of novel actions. Individuals with AS imitated as frequently and as accurately as typical young children in response to live demonstrations; but they imitated less frequently and less accurately in response to video-recorded demonstrations. Further, imitation was modulated by whether the demonstrator was socially engaging or emotionally neutral in the AS group, while this modulation was not present in the comparison group. Individuals with higher mental age imitated more frequently and more accurately across groups. Imitation performance in AS appears to be more modulated by the social context compared to typical infants and young children with similar mental age, possibly reflecting an enhanced drive for social engagement. A socially engaging instructional style might facilitate imitative learning in this population.

## Introduction

Angelman syndrome (AS) is a rare neurodevelopment disorder (estimated incidence 1 in 12.000–20.000^1^) caused by disruption of the maternally-inherited UBE3A gene, most commonly due to a large deletion of the chromosome 15q11.2-q13 region, or to a pathogenic variant of the maternal copy of the UBE3A gene, paternal uniparental disomy, or imprinting defect^2,3^. The clinical phenotype in AS is characterized by severe to profound intellectual disability, ataxic gait, epilepsy, and minimal or absent speech, with between 71–90% of individuals with AS never or rarely producing any speech^4,5^. Psychomotor delay is usually evident within the first year of life^6^, with an upper mental age limit of 24–30 months. Additional behavioral and neuropsychological characteristics frequently associated with AS include feeding problems, frequent laughing and smiling, sleep disturbances, hyperactivity, and short attention span^7–10^.

Communication challenges observed in AS extend beyond verbal skills and encompass difficulties in social-pragmatic and social-cognitive processes, such as joint attention and dyadic engagement^11^, making it difficult for children with AS to successfully engage in sustained social exchanges. Alvares and Downing^12^ reported that in a sample of 20 children with AS, only 50% of them maintained eye contact during a communicative exchange and only 20% intentionally gained their partner’s attention prior to signing. Penner and colleagues^13^ found that only one of seven adults with AS was able to jointly attend and act on objects with another person and to participate in a turn taking exchange.

Despite these challenges, children with AS manifest a high desire to communicate with others^14,15^, and show an atypically elevated frequency of laughing and smiling behaviors, particularly during early childhood and in the context of highly engaging social interactions^16^. This “happy disposition”, however, tends to decrease with age^17^. The co-existence of social communication difficulties with a distinctive drive for social engagement in children with AS provides a puzzling phenotypic expression, with important implication for treatment and research on typical and atypical social communication development. However, research in this area remains limited, partly due to methodological challenges^18^, including difficulties with designing evaluation measures and conducting objective assessments that are adapted to the presence of severe cognitive impairment, tremors and ataxia, short attention span, motivational limitations and lack of verbal skills.

An important dimension of social-communicative functioning where information in AS is lacking is imitation. Imitation is a critical skill for social learning and social engagement^19–24^, emerging during the first year of life and serving important social-communicative and learning functions. In both typical and atypical development, imitation is longitudinally associated with cognitive and language skills^25–29^, and concurrently associated with joint attention^30,31^, affect sharing^32–34^, empathy^35,36^, and the ability to cooperate^37^.

Additionally, research has shown that imitation performance in typical children and those with neurodevelopmental disorders is modulated by social factors. For example, a recent study^38^ has shown that children with Williams syndrome, but not children with autism spectrum disorder (ASD), tend to spontaneously imitate socially engaging models more frequently than “emotionally neutral” ones, a phenomenon thought to reflect the social and affiliative motives that underlie spontaneous imitation^39^. The social modulation of imitation has been also highlighted by studies documenting the so called “transfer deficit” – also known as “ “video deficit effect^40^”, whereby typical infants and toddlers imitate actions presented through videos with reduced accuracy and frequency when compared with a live model^41–43^, possibly due to the lack of opportunity for contingent interaction^44^. Additional research shows that infants perform tasks differently—and better—when they are in the presence of another person, as their imitation appears to be enhanced by the mere presence of somebody who interacts with them^45^.

Despite the relevance of imitation for social-cognitive development, its interplay with social engagement and motivation, and the availability of paradigms designed to evaluate this skill in children with severe communicative and cognitive impairments, research on imitation in AS is extremely limited. An observational study on 11 cases^11^ documented that only AS children with milder symptoms were able to imitate motor actions such as clapping and waving. More recently, a study designed to assess memory, imitation and motor performance in 12 children with AS^46^ documented the presence of severe difficulties in imitation of new motor actions. None of these studies used comparison groups to examine the specificity of imitation difficulties. Additionally, it is possible that the difficulties in imitation in these studies reflected challenges in understanding verbal instructions in the imitation tasks. Further, no examination was conducted on how imitation performance was modulated according to the social context—an important factor in the context of AS, given the research showing the strong influence of the social context on social behaviors in this population. Finally, previous literature on AS did not operate a distinction between accuracy of imitation (the degree to which the imitated action matches the model) and propensity of imitation (the frequency to which children imitate in the absence of specific instructions). This is an important distinction, as these two dimensions are associated with different social and cognitive processes in typical and atypical populations^47^. Given the drive for social engagement documented in the AS population, information on social factors that facilitate imitation can provide critical insight on intervention, as effective behavioral and educational interventions for children with neurodevelopmental disorders use imitation as a critical learning tool^39,48^.

In order to address these knowledge gaps and open questions, the current study provides the first controlled examination of imitation in children with AS, using a novel experimental paradigm designed to test how imitation varies according to the social context.

Given the Angelman syndrome-specific combination of strengths and weaknesses in the social domain and evidence for social modulation of behavior, we focused our examination on how imitation in this group is moderated by different levels of social engagement with the person demonstrating the to-be-imitated actions. Unlike previous literature on the topic, which mostly focuses on deferred imitation, the current study examines immediate imitation in order to maximize experimental control and avoid the potential confounding factor of memory abilities.

The following research questions were addressed:

1. Do individuals with AS differ from mental age—matched children with typical development in their tendency to imitate spontaneously (frequency of imitative responses) and their ability to imitate (accuracy of imitative responses)?
2. Do individuals with AS and those with typical development show different imitative behaviors in response to socially engaging versus non-socially engaging models?
3. Do individuals with AS and those with typical development show different imitative behaviors in response to live versus video-recorded demonstrations?
4. Are imitation skills associated with mental age in AS?

## Results

### Study 1: imitation live (3D)

We first compared overall imitation frequency and imitation accuracy between the two groups considering all the 8 trials (4 in the playful condition and 4 in the neutral condition) collectively. Actions were imitated by the Angelman syndrome group (ASG) in 60.65% of cases (95% IC 48.94–72.36), with an accuracy level of 37.7% (95% IC 27.61–47.87). The comparison group (CG) imitated 65.6% (95% IC 48.45–87.78) of actions across the 8 trials, with a level of accuracy of 46.7% (95% IC 32.05–61.28). Results of a beta-binomial regression showed no significant differences between the ASG and the CG in the frequency (p = 0.189) or accuracy (p = 0.207) of imitation.

**Figure 1:**
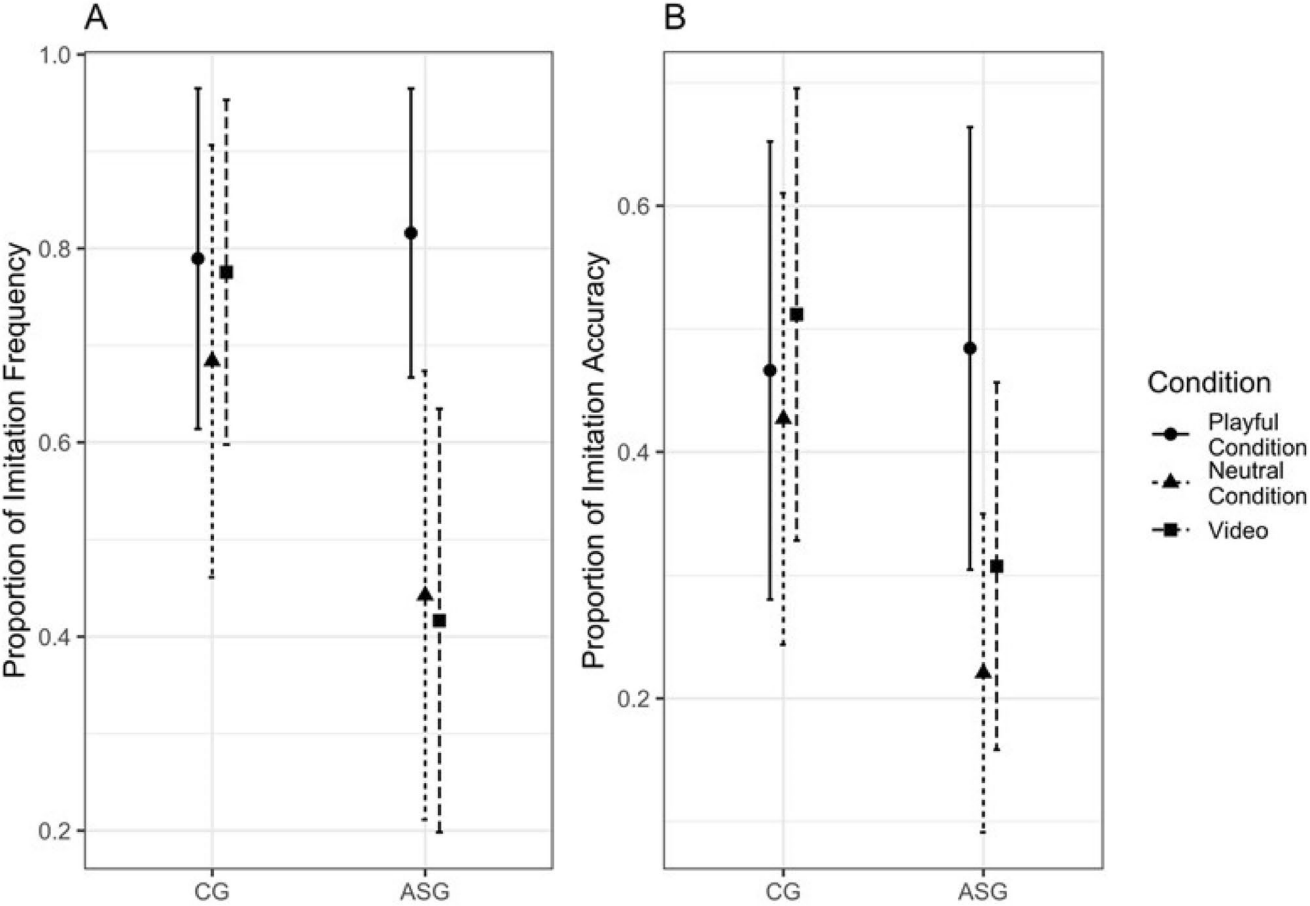
Bar-plots of the proportion of imitation frequency (**A**) and accuracy (**B**) by group and condition estimated through a beta-binomial mixed effect model.

When the two conditions were considered separately (i.e., trials were stratified according to the level of social engagement expressed by the demonstrator, namely playful and neutral conditions), the ASG imitated 73.9% (95% CI 61.51–86.32) of the actions in the playful condition, while the frequency of imitation in response to the neutral condition was 45.65% (95% CI 32.35–58.95). Imitation frequency in the CG was 69.05% (95% CI 51.09–87.01) in response to the playful condition, and 61.9% (95% CI 44.42–79.39) in response to the neutral condition. Results of the beta-binomial mixed effect model indicated that imitation frequency was similar in the ASG and the CG across the neutral and the playful conditions (p = 0.157 and p = 0.818 respectively) , but there was a significant increase in the frequency of imitation in the playful condition compared to the neutral one in the ASG (p = 0.001), which was not observed in the CG (p = 0.479) (Fig. 1A).

The ASG imitated more accurately in response to the playful condition (accuracy 47.82%—95% CI 35.88–59.76) compared to the neutral condition (accuracy 27.88%—95% CI = 17.14–36.6; p = 0.007). The CG imitated the playful and neutral models to a similar level of accuracy (accuracy level in the playful condition = 48.21%—95% CI = 33.23–63.19; accuracy level in the neutral condition = 45.38%—95% CI 30.18–60.58; p = 0.783). The beta-binomial mixed effect model showed that imitation accuracy in the neutral condition was lower in the ASG compared to the CG at a marginally significant level (p = 0.073) while imitation accuracy in the playful condition was similar in the ASG and the CG (p = 0.891). In the ASG, imitation accuracy was higher in response to the playful compared to the neutral condition (p = 0.001), while this was not the case in the CG (p = 0.864) (Fig. 1B).

### Study 2—imitation from a screen (2D)

In response to video-recorded demonstrations of actions, the frequency of imitation in the ASG was 45.65% (95% CI 29.22–62.09), while accuracy was 35.87% (95% CI 20.77–50.97). The CG imitated 69% (95% CI 51.66–86.34) of the observed actions, with a total accuracy 53.24% (95% CI 38.87–67.61). Results of the beta-binomial mixed effect regression model indicated that imitation frequency was significantly lower in the ASG than in the CG (ASG—CG − 23.3; 95% CI − 46.5, − 1.3; p = 0.023) and a similar trend was observed for imitation accuracy, although the difference did not reach statistical significance (ASG—CG − 17.4; 95% CI − 36.6, 1.3; p = 0.096).

### Different imitative behaviors in response to live versus video-recorded demonstrations

When considering imitation frequency, the ASG imitated more frequently live playful models, compared not only to live neutral models, as reported in the analyses above, but also to video-recorded playful models (p < 0.001). A similar pattern was found with regard to imitation accuracy when comparing live versus video-recorded playful models (p = 0.029). The CG did not show significant variations in imitation frequency (p = 0.982) or accuracy (p = 0.818) in response to live versus video-recorded playful models (see Fig. 1; Table 1).

**Table 1:** Participants’ characteristics.

	ASG Mean; SD (IC 95%)	CG Mean, SD (IC 95%)
Chronological age (months)	142; 110.79 (94.70–190.52)	23.9; 4.87 (21.64–26.07)
Gender (M;F)	(13; 8)	(12; 11)
**Griffiths mental developmental scales—Extended Revised (expressed in mental age—months)**		
**Subquotients**		
Motor	19.3; 8.91 (15.44–23.16)	24.3; 4.83 (22.14–26.55)
Personal–social	21.38; 11.49 (16.41–26.35)	25.4; 5.6 (22.87–27.97)
Language	13.27; 4.31 (11.41–15.14)	24.7; 5.17 (22.31–27.01)
Hand–eye coordination	19.99; 10.81 (15.32–24.7)	24.3; 4.49 (22.27–26.36)
Performance	19.75; 12.13 (14.5–25)	24.4; 6.66 (21.37–27.43
Total quotient	20.1; 9.86 (15.84–24.42)	24.6; 4.65 (22.57–26.81)

### Frequency and accuracy of imitation in association to mental age

We tested the association between mental age (MA) and imitation performance across all trials in study 1 (playful plus neutral actions), and found a positive trend showing higher imitation frequency and accuracy in participants with higher MA across groups. Such trend was statistically significant only for the accuracy score in the CG (p = 0.004) and for both frequency and accuracy scores in the ASG (p = 0.026 and p = 0.003 for frequency and accuracy scores, respectively—see Table 2).

**Table 2:** Results of the effect of mental age (MA) in the Angelman syndrome group (ASG) and the comparison group (CG) on imitation frequency and accuracy in study 1 (3D) and the effect difference between the two groups.

	Mental age CG Effect (CI); p-value	Mental age ASG Effect (CI); p-value	Mental age ASG–CG Effect (CI); p-value
Imitation frequency total live	1.00 (− 0.14, 2.14); 0.083	0.63 (0.08, 1.18); 0.026	− 0.37 (− 1.63, 0.89); 0.555
Imitation accuracy total live	1.25 (0.43, 2.06); 0.004	0.57 (0.20, 0.94); 0.003	− 0.68 (− 1.57, 0.21); 0.131
Imitation frequency live neutral condition	1.26 (− 0.45, 2.97); 0.146	0.77 (0.04, 1.51); 0.039	− 0.49 (− 2.35, 1.36); 0.601
Imitation accuracy live neutral condition	1.37 (0.29, 2.45); 0.014	0.60 (0.12, 1.08); 0.015	0.77 (− 0.41, 1.94); 0.200
Imitation frequency live playful condition	1.85 (− 0.08, 3.79); 0.061	3.65 (1.04, 6.27); 0.007	1.80 (− 1.40, 5.00); 0.267
Imitation accuracy live playful condition	1.50 (0.39, 2.60); 0.009	1.07 (0.43, 1.72); 0.001	0.42 (− 0.86, 1.70); 0.513

A beta-binomial regression was used for Total scores in study 1 for imitation frequency and accuracy, while a beta-binomial mixed effect model was applied when considering live playful, neutral and video recorded playful conditions.

Considering imitation performance separately in the playful and neutral conditions, we found a positive significant association between the MA and imitation frequency for the ASG across the neutral (p = 0.039) and playful condition (p = 0.007). No significant associations were found in the CG between MA and imitation frequency across conditions (see Table 2).

**Figure 2:**
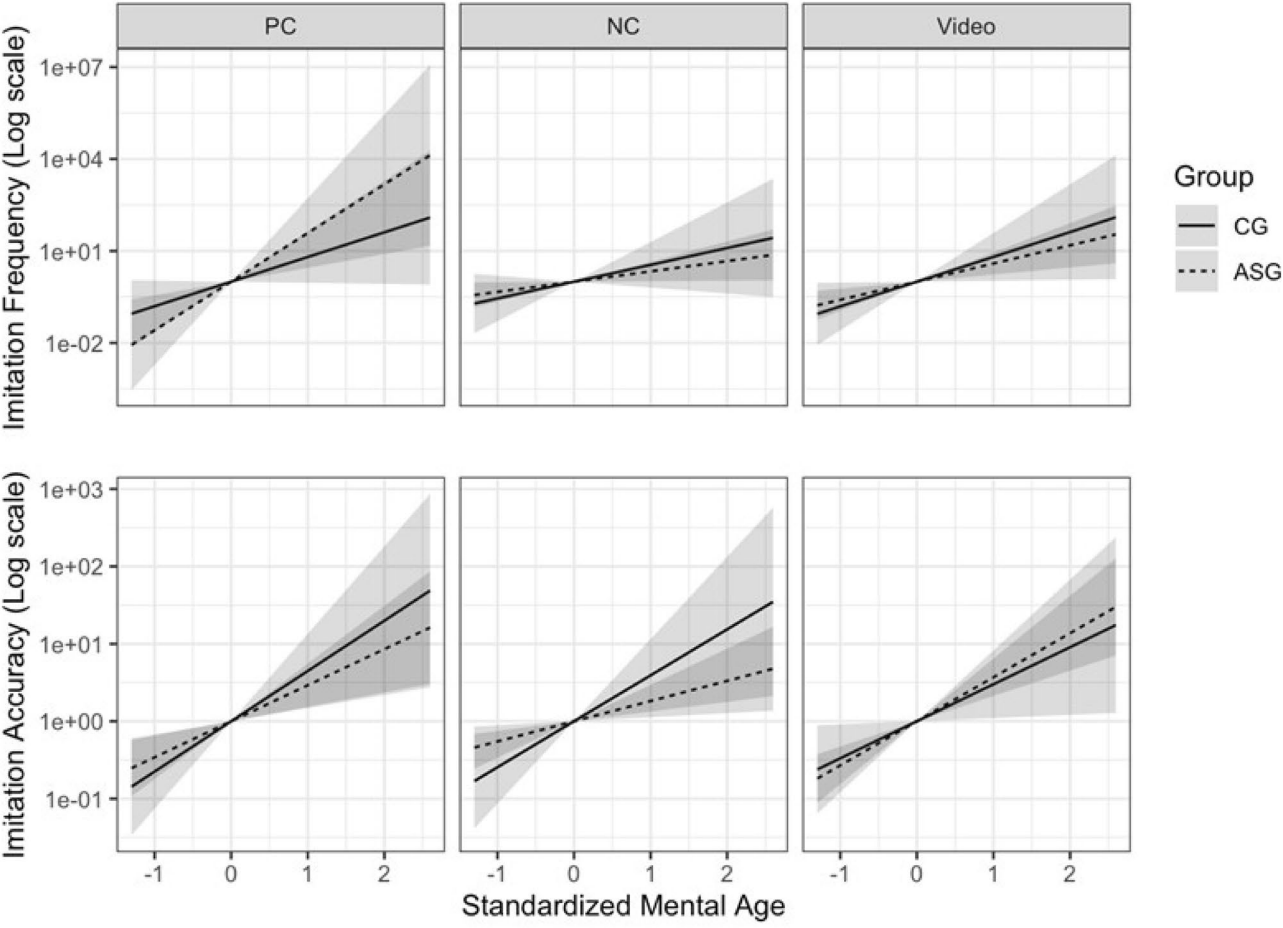
Effect of mental age on imitation frequency and accuracy by group and condition estimated through a beta-binomial mixed effect model. A log transformation is applied for the y axis.

As illustrated in Fig. 2, the association between MA and imitation accuracy was positive and significant for both the ASG and the CG across conditions (Neutral Condition: p = 0.015 in ASG, p = 0.014 in CG; Playful Condition: p = 0.001 in ASG, p = 0.009 for CG).

When comparing the two groups no significant difference was found in the association between MA and imitation frequency/accuracy across both playful and neutral conditions (see Table 2, Fig. 2).

Finally, we found a positive significant association between MA and imitation frequency and accuracy in study 2 (imitation from a screen—2D) across both the ASG (p = 0.001 for frequency, p < 0.001 for accuracy) and the CG (p = 0.042, p = 0.032 for imitation frequency and accuracy respectively). When comparing the two groups, no significant difference was found in the association between MA and both imitation frequency and accuracy (see Table 3).

**Table 3:** Results of the effect of mental age (MA) in the Angelman syndrome group (ASG) and the comparison group (CG) on imitation frequency and accuracy in study 2 (2D) and the effect difference between the two groups.

	Mental age CG Effect (CI); p-value	Mental age ASG Effect (CI); p-value	Mental age ASG–CG Effect (CI); p-value
Imitation frequency video recorded playful condition	1.86 (0.07, 3.65); 0.042	1.37 (0.53, 2.20); 0.001	− 0.50 (− 2.46, 1.46); 0.617
Imitation accuracy video recorded playful condition	1.10 (0.10, 2.11); 0.032	1.31 (0.75, 1.87); < 0.001	− 0.21 (− 1.36, 0.94); 0.720

A beta-binomial mixed effect model was applied when considering live playful, neutral and video recorded playful conditions.

Additionally, we found an association between imitation performance in study 2 (imitation from a screen—2D) and chronological age across both imitation frequency and the accuracy for ASG, showing older children performing better than younger ones (p = 0.033 and p = 0.017, respectively; p-values are obtained from a beta-binomial regression as conducted for MA).

Finally, we examined whether results of our analyses changed when only children in the ASG who had presented deletion of the chromosome 15q11.2-q13 region were included in the analyses (AS-deletion; n = 16). Results, which are reported in the Supplementary information 1, are substantially overlapping with those obtained when the entire ASG is considered.

## Discussion

In this study, we examined for the first time spontaneous imitation in individuals with AS in comparison to typical children with a similar mental age. Imitative performance was evaluated in response to three conditions (1) live demonstration by a socially engaging (playful) model, (2) live demonstration by an emotionally neutral model, and (3) video-recorded demonstration by a socially engaging model. The rationale for the use of these conditions was based on the importance of socially-motivated and socially-modulated imitation in typical and atypical development, and the lack of previous research on these factors in the AS population.

Results showed that individuals with AS imitated as frequently and as accurately as typical children with similar mental age in response to a live demonstration of simple actions on objects.

However, imitation was more modulated by the level of social engagement expressed by the demonstrator in the ASG compared to the CG. In response to a video-recorded demonstration, individuals with AS imitated less frequently and less accurately than those in the typical CG. Additionally, individuals with AS imitated more frequently and more accurately in response to the live model compared to the recorded video, while this modulation was not present in the CG, where accuracy and frequency of imitation were similar in the two conditions. This pattern of results suggests that social factors, including the playfulness and the live interaction of the demonstrator modelling the actions, improve imitation performance in individuals with AS. Research and clinical implications are discussed in the following sections.

Our results suggest that individuals with AS are able to imitate simple actions, and their overall imitation performance is similar to that shown by younger typical children with similar mental age. These findings differ from previous reports of imitation difficulties in AS^11,13,14,46,49,50^. For example, using parental reporting^14,49^ previous research has documented that individuals with AS were able to communicate across pragmatic contexts, including rejecting and protesting, and requesting and commenting, but imitation (together with requesting information) was the least developed area of communication. In Didden et al.’s studies^14,46^, individuals with AS were reported to be unable to imitate non-verbal communicative behaviors, as compared to other individuals with severe and profound intellectual disability. However, this conclusion was based on caregivers’ reports in response to a questionnaire investigating language skills. Therefore, conclusions of these studies might differ from the current study due to methodological differences. Another study^44^ tested individuals with AS using an observational protocol involving simple motor actions without objects, reporting poor imitation performance. However, unlike our experimental protocol, the imitation task was administered at home, with parents acting as actors. Differences in the setting and the nature of to-be-imitated actions (actions without objects versus actions on objects) might explain the different results between this and the current study. Finally, consistent with results of the current study, Jolleff and Ryan^11^ documented that 5 out of 11 participants with AS could imitate simple actions, such as clapping or waving, using items derived from the Preverbal Communication Schedule of Kiernan^50^. These previous studies involved methodological limitations, including lack of comparison groups and lack of control for mental age, that complicate interpretation of previous literature in this area.

Although we found no overall imitation deficit in AS, we found differences in how imitation was modulated by the social context in AS compared to typical young children. Specifically, individuals with AS appeared to be more influenced by two social factors, namely the playful versus neutral affect the demonstrator showed, and physical presence of a demonstrator versus a video-recorded demonstration. These data seem to be consistent with previous research documenting an enhanced drive for social engagement in children with AS. For example, a study by Oliver and colleagues^51^ documented that social behaviors such as smiling and laughing in AS are modulated by the presence/absence of engaging adults. Interestingly, these results highlight a phenotypic dissimilarities between children with AS and those with autism spectrum disorder (ASD), whose imitation performance, as reported in previous research, appears to be less modulated by social factors such as playfulness^38^ (although see Nadel, 2014^52^, and Ingersoll et al., 2017^48^, for evidence of imitation enhancement in ASD following reciprocal imitation intervention strategies). The different imitation performance in the AS group in response to live versus video-recorded demonstrations appears to reflect the “transfer deficit” phenomenon previously discussed in the context of the literature on typical development. Surprisingly, however, we did not observe “transfer deficit” in typical children in the present study, as their imitation performance was similar across live and video-recorded presentations. This unexpected finding might reflect the very simple nature of the to-be-imitated actions included in our paradigm.

Overall, our results suggest that in individuals with AS the role of social engagement in driving imitative responses is more relevant than in infants and toddlers with similar mental age, and children with other neurodevelopmental conditions such as ASD. These findings may have direct implications for treatment, whereby imitation might be targeted in the context of socially engaging activities with a lively and animated adult, rather than less socially engaging strategies such as video-modeling.

Most of our participants with AS had a mental age between 10 and 24 months, consistent with previous literature on cognitive level in this syndrome^6^, and their imitation performance in response to the demonstration of simple one-step actions was in line with the performance expected for children within 24 months^53^. The comparison with the much younger typically developing participants suggests that the approximately 10 years of additional experience of the social world in the AS group had a limited impact on their imitation performance. Additionally, there was a substantial association between mental age and imitation performance across conditions in the AS group, suggesting that children who were developmentally more advanced imitated more frequently and more accurately. A similar pattern was found in the comparison group. Therefore, both cognitive and social factors appear to play an important role in children with AS. Although this is generally the case for all children, our results are consistent with the notion of a syndrome-specific elevated responsivity to the reinforcing properties of social interaction in AS, at least during childhood^17^. This atypical social phenotype, and its interplay with the cognitive impairments, might explain the heightened social and cognitive modulation of imitation in the AS group compared to the typical group. However, the simple nature of the to-be-imitated actions and the homogeneous cognitive level in the comparison group might have contributed to the lack of social modulation in the typically developing comparison group.

Other limitations in the current study that should be acknowledged are listed in the following section. First, we did not systematically manipulate the motor complexity of the tasks, and therefore our conclusions are limited to the imitation of simple one-step actions. Second, the to-be-imitated actions modeled in study 1 were different from the ones presented in study 2, in order to prevent learning/familiarization over time. Therefore, it is possible that differences in features of the modeled actions contributed to different performances in the live versus video conditions in AS. However, extreme care was taken in selecting actions with similar motor complexity, and the similar imitation performance in the typical group across live and video demonstrations suggest that the use of different actions may not have influenced the current findings. Further, there was a wide range in chronological age in the AS group, due to the recruitment difficulties associated with the low prevalence of this syndrome. However, we were able to mitigate this limitation by successfully matching children by mental age across groups. Additionally, given the previous literature on reduced eye-contact in AS, it cannot be excluded that the group differences in the video-recorded demonstration condition reflected reduced attention toward the demonstration in children with AS. This limitation should be addressed by future research using eye-tracking techniques to examine whether modulation of imitation in different conditions in AS is mediated by differences in attentional engagement in response to live versus video-recorded demonstrations. Social engagement measures could provide additional insight on how imitation performance in AS is influenced by the interplay between social reciprocity and cognitive factors at different ages and across the spectrum of cognitive functioning in this population. The consideration of social engagement in non-imitative contexts and how it changes with age is particularly important given previous research showing age-related changes in sociability of children with AS in different social contexts^16,17^. A fine-grained characterization of language skills would also provide further insight on factors related to imitation in this population. Another factor that should be examined in future research is participants’ previous experience with 2D material, as the child history of engagement with video-presented stimuli might affect their propensity to imitate in response to live versus video-recorded demonstrations. Finally, in order to make the experimental paradigm suitable for severely affected children with AS and avoid fatigue and non-compliance, it was necessary to limit the number of trials in each task. Despite these limitations, to our knowledge this is the first controlled study focusing on imitation in individuals with AS, thus providing new insight on the social and cognitive factors associated with imitation in this under-studied population.

In conclusion, our study demonstrated that individuals with AS imitate simple motor actions with objects to the same frequency and accuracy than typically developing children with similar mental age. Individuals with more advanced mental age imitated more frequently and more accurately. Imitation performance in AS appears to be more modulated by the social context compared to typically developing children, possibly reflecting an enhanced drive for social engagement. It is possible therefore that a socially rich and playfully engaging instructional style might facilitate imitative learning in this population. This is particularly important in the context of the current trend toward telehealth approaches to intervention for children with neurodevelopmental disorders^54^. Such approaches might be less beneficial for children with AS compared to in-person delivery approaches, especially for strategies involving imitation. This treatment implication should be empirically substantiated by future research.

## Methods

### Participants

Participants in the Angelman syndrome group (ASG) included individuals with a genetic-confirmed diagnosis of AS. Additionally, the study included a comparison group (CG) of infants and young children with typical development and a similar mental age (MA).

The ASG included 23 participants (11 females, mean chronological age of 11.11 years, SD = 9.3 years, 95% CI 7.11–15.10 years; range = 1–37 years), recruited through our children hospital unit and through the Italian Angelman Syndrome Organization (OR.S.A.).

Inclusion criteria for the ASG involved a molecular confirmed diagnosis of AS, visual acuity higher than 3 dec., well-controlled epileptic seizures, Italian as the primary language spoken at home, absence of uncorrected hearing or vision impairment, and absence of major medical problems beyond AS syndrome. As to the genetic profile, 16 individuals presented a UBE3A deletion, 5 a UBE3A mutation, 1 child had a uniparental disomy and 1 an imprinting defect. Nineteen individuals suffered from epilepsy and 18 of them used antiepileptic therapy, with well-controlled seizures. As reported in the Table 1, their MA, as measured through Griffiths Mental Developmental Scales—Extended Revised (Griffiths ER)^55,56^, was 20.1 months (SD 9.9 months, 95% CI 15.8–24.4 months; range = 11–41 months). All the participants with AS attended or had attended school within mainstream educational settings with support from special education teachers. They all lived at home with their parents.

Children in the CG were recruited among patients referring to our children hospital to attend orthopedic, pediatric or surgical visits. The following inclusion criteria were used in the study: absence of a known history of medical conditions, psychomotor development within the normal range (Griffiths ER Developmental Quotient equal or higher than 100), absence of language deficits or language delay, a good understanding of the Italian language and absence of any sensory impairment. The recruitment procedures in the CG involved information to the children and their families at the end of their outpatient visit. The families that consented to have their child participate in the study were administered an anamnestic data interview focused on pre-peri-post-natal events and psychomotor development, including motor, language, and social development. Children who satisfied inclusion criteria were administered the Griffiths ER. Those who reached a standard score equal or higher than 100 were administered the experimental imitation paradigm. The CG included 21 infants and young children, 13 males and 8 females, with a mean chronological age of 23.9 months (SD = 4.9 months, 95% CI = 21.6–26.1 months; range = 18–36 months), similar to the mental age of the ASG (p = 0.229). Participant characteristics are detailed on Table 1.

### Procedures

This study was approved by the institutional review board of ASST Spedali Civili of Brescia (Comitato Etico di Brescia, ID number: ASET-NP 2890). All study procedures were performed following the relevant guidelines and regulations. Informed consent was obtained from all participants’ parents or legal guardians.

**Figure 3:**
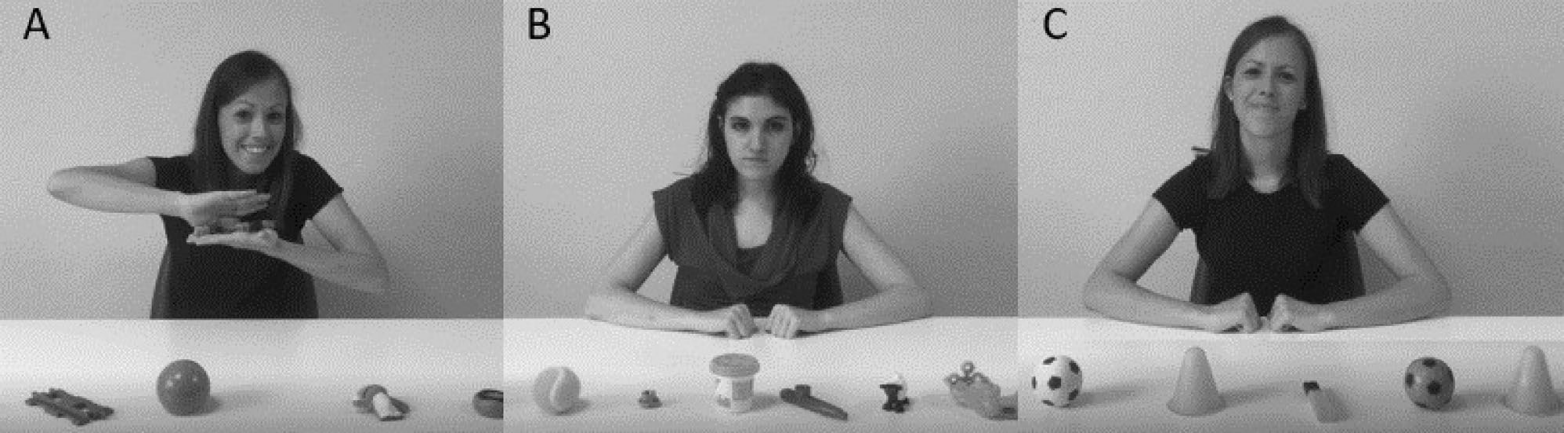
Live playful (**A**), live neutral (**B**) e video-recorded playful (**C**) models in studies 1 and 2.

Participants in the study were tested across two testing sessions within up to 15 days in a quiet room of our Unit. The first visit included the administration of the Griffiths ER. Subsequently, participants across groups who satisfied all the inclusion criteria were administered a 30-min long experimental imitation paradigm in the second visit. Experimental procedures were based on the imitation tasks described in Vivanti et al.^57^ (Study 1) and in Vivanti et al.^38^ (Study 2). The tasks were designed to examine imitative performance in response to different social factors, including the presence of socially engaging versus socially neutral models (Study 1), and live/interactive versus video-presented models (Study 2—Fig. 3). The order of presentation of studies 1 and 2 was randomized, in order to avoid learning effects or habituation to models’ faces.

### Study 1—imitation live (3D)

Subjects were encouraged to sit at a table opposite the experimenter and were presented with a set of eight objects. There were four imitation trials, involving four different sets of eight objects. Each trial consisted of a “playful condition” and a “neutral condition”.

In the playful condition, the experimenter, after obtaining the child’s attention, performed an arbitrary action on one of the objects (for example, placing a little ball on the shoulder) in a playful, socially engaging way, which included emotional expressions of surprise and happiness, as well as lively, animated body language. After the demonstration, the experimenter put the object back on the table together with the other objects. No verbal instruction was provided, and participants’ spontaneous behavior in response to the demonstration in the 15 s following the demonstration was observed and recorded for later coding.

In the neutral condition, a second experimenter demonstrated another action on a different object of the same set, but in a neutral way, i.e., without displaying facial or bodily emotions. In both conditions, the experimenter alternated gaze between the object and the child. Each experimenter played the same role, playful or neutral, across the experiment. No verbal communication was used during the demonstration. Similar to the playful condition, the child’s behavior in response to the demonstration in the subsequent 15 s was recorded for later coding. In each trial the neutral condition was followed by the playful condition with the same set of objects, then the next trial was started with a new set of objects.

The rationale for not providing explicit instructions and not using verbal communication was to avoid the confounding factor of understanding verbal instructions, given the severe communication difficulties in the ASG. This procedure has been used in previous research involving children with severe neurodevelopmental disorders, with data indicating that the paradigm used in the study was successful in eliciting spontaneous imitation in the absence of specific verbal instructions^57,58^. The rationale for using arbitrary actions rather than the action most commonly associated with the object (e.g., placing a container on the shoulder instead of opening the container) was to ensure that the child’s actions reflected imitation, rather than being merely triggered by the objects’ affordance.

A third experimenter was present in the room during testing, and scored all trials in the moment. Sessions were videotaped for later independent coding and inter-rater reliability analyses. The camera that recorded each session was placed on the side of the table on which the study was conducted, so that it could capture both the examiner’s demonstrations and participants’ imitation responses. An independent coder, who was naïve to the aims of the study and to the third experimenter’s scoring, scored each videotaped session using operational definitions for correct responses based on Vivanti et al.^57^ A simple yes/no (1/0) criterion was used to code whether participants spontaneously imitated each modelled action. A total frequency score was obtained for each participant by calculating the proportion of imitated actions out of the total imitation opportunities. Accuracy performance was coded using the three-point Likert scale coding procedure based on Vivanti et al.^57^, whereby participants were assigned 2 points if they imitated the action performed by the experimenter, 1 point if they operated on the same object used by the experimenter but not the same action, 0 points for any other response. The rationale for attributing a score of 1 even if the action performed by the participant was different from the action performed by the demonstrator was to capture children’s propensity to act on the same object involved in the demonstration. The child’s engagement with the same object used by the demonstrator rather than an unrelated object reflects the social learning phenomenon often described in literature as “social enhancement”, which is considered to be a “primitive” form of social learning and a precursor of imitation^39^. Thus, in the context of the cognitive delay that characterizes AS, our coding system was designed to capture the difference between the complete lack of response to the demonstration versus responses that, while not qualifying as proper imitation, reflected a rudimentary form of imitation (i.e., imitating the behavior of acting on the object X as opposed to object Y).

A total “imitation accuracy” score was calculated by summing the scores assigned for each item and then converting the sum into a percentage score.

Inter-rater reliability was calculated on 20% of the videos using Cohen’s Kappa, with results showing 100% agreement for imitation frequency and 87.3% for imitation accuracy. The scores from the experimenter who coded from video, who was blind to the study aims, were used in the analyses.

Additionally, the coder examined whether demonstrations were administered correctly, and found this to be the case for across all trials. Therefore, all trials were retained in the analyses.

### Study 2—imitation from a screen (2D)

Participants were shown a series of six video stimuli (7 s each) through a computer monitor while seated in a comfortable chair, 60 cm from the computer monitor in front of a small table. In each video, the same female demonstrator performed a simple action involving one of six objects placed on the table in front of her. Similar to study 1, each demonstration involved an arbitrary action (e.g., placing a cone on the palm of the hand). The demonstrator displayed a playful, positive affect throughout the demonstration, and alternated gaze between the object and the child. Two different sets of objects were used in the 6 videos. Each video involved a different action on a different object. Therefore, each to-be-imitated action was demonstrated only once. Additionally, the demonstrated actions were different than the one used in study 1 to avoid learning effects. The presentation of the video stimuli was arranged in the same fixed random order across participants in the two groups.

The same objects used in the demonstration were placed on the small table in front of the child after each video. Following the same procedures as in study 1, no explicit direction was given, and participants’ spontaneous behavior on the objects in response to the video-demonstration was recorded for coding purposes.

The same coding procedures of study 1 were applied in study 2. An experimenter presented in the room scored all trials in the moment. Sessions were videotaped for later independent coding for inter-rater reliability. An independent scorer, who was naïve to the aims of the study and to third experimenter’s scoring, scored each videotaped session using the operational definitions for correct responses detailed above. Inter-rater reliability was calculated on 20% of the videos using Cohen’s Kappa, with results showing 100% agreement for imitation frequency and 86.6% for imitation accuracy. The scores from the experimenter who coded from video, who was blind to the study aims, were used in the analyses.

### Statistical analyses

The imitation performance of the ASG and CG in response to the different conditions across both studies was computed in terms of percentage scores (proportion of imitated actions out of the total number of demonstrated actions for imitation frequency, and percentage of accuracy for imitation accuracy) and 95% CI. A generalized mixed effect model with beta-binomial family was used to test for the difference in proportion of imitation frequency and accuracy across playful and neutral condition and study 2 total score in the two groups. An interaction term between groups and mental age (MA for the ASG and chronological age for the CG; from now on MA) was then introduced in the regression models to test for the effect and the difference in the association between mental age and accuracy and imitation frequency (across both the playful and neutral conditions) in the two groups. A beta-binomial regression was also applied when the total imitation frequency and accuracy were considered for study 1. MA was standardized when the interaction with the group variable was included in the regression models. We considered two-sided alpha below 0.05 as statistically significant for all tests and regression models. The Tukey’s adjustment was applied to adjust for multiple comparisons. All statistical analyses were performed with R 4.0.0^59^.

## Supplementary information

Supplementary file 1

## Data Availability

The datasets used for the current study are available from the corresponding author on request.
