# Supplementary material for: Imitation in Angelman syndrome: the role of social engagement
Source: Sci Rep. 2020 Oct 2;10:16398. doi: 10.1038/s41598-020-72079-3 (PMC7532435; doi:10.1038/s41598-020-72079-3)
Supplement: Supplementary file 1 [file 41598_2020_72079_MOESM1_ESM.pdf]

IMITATION IN ANGELMAN SYNDROME: THE ROLE OF SOCIAL ENGAGEMENT

Serena Micheletti[1*], Giacomo Vivanti[2], Stefano Renzetti[3], Paola Martelli[1], Stefano Calza[3], "Imitation in Angelman" Study Group, Elisa Fazzi[1,4]

SUPPLEMENTARY INFORMATION

We repeated the same analysis reported in the Results section, including only individuals with AS who presented a deletion of the chromosome 15q11.2-q13 region (AS-deletion group - N= 16).

Study 1 - Imitation live (3D)

We repeated the same analysis reported in the Results section, including only individuals with AS who presented a deletion of the chromosome 15q11.2-q13 region (AS-deletion group - N= 16). When the two conditions were collapsed the AS-deletion group imitated 50% of the actions (95% CI 36.68-63.32) with a percentage accuracy of 26.17% (95% IC 19.05-33.29), which was a significantly lower performance compared to the CG, (p=0.018 for imitation frequency and, p=0.005 for imitation accuracy).

When the two conditions were considered separately we saw the same pattern of results that we found when the entire ASG included in the analyses. The imitation performance in the AS-deletion group was significantly higher in response to the Playful Condition as compared to the Neutral Condition (p=0.007 for imitation frequency and p=0.033 for imitation accuracy), a pattern that was not observed in CG. Specifically, with regard to imitation frequency the AS-deletion group imitated 64.06% of the demonstrated actions in the Playful Condition (95% CI 48.7-79.4) versus 35.95% (95% CI 21.37-50.51) in the Neutral Condition. CG performed better in NC compared to ASG (p=0.035). With regards to imitation accuracy, imitation performance in the AS deletion was 34.38% (95% CI 25.78-42.97) in the Playful Condition versus 17.97% (95%CI 10.69-25.25) in Neutral Condition. Also in this case CG showed a higher imitation accuracy than ASG in NC (p=0.008). See Figure 1S.

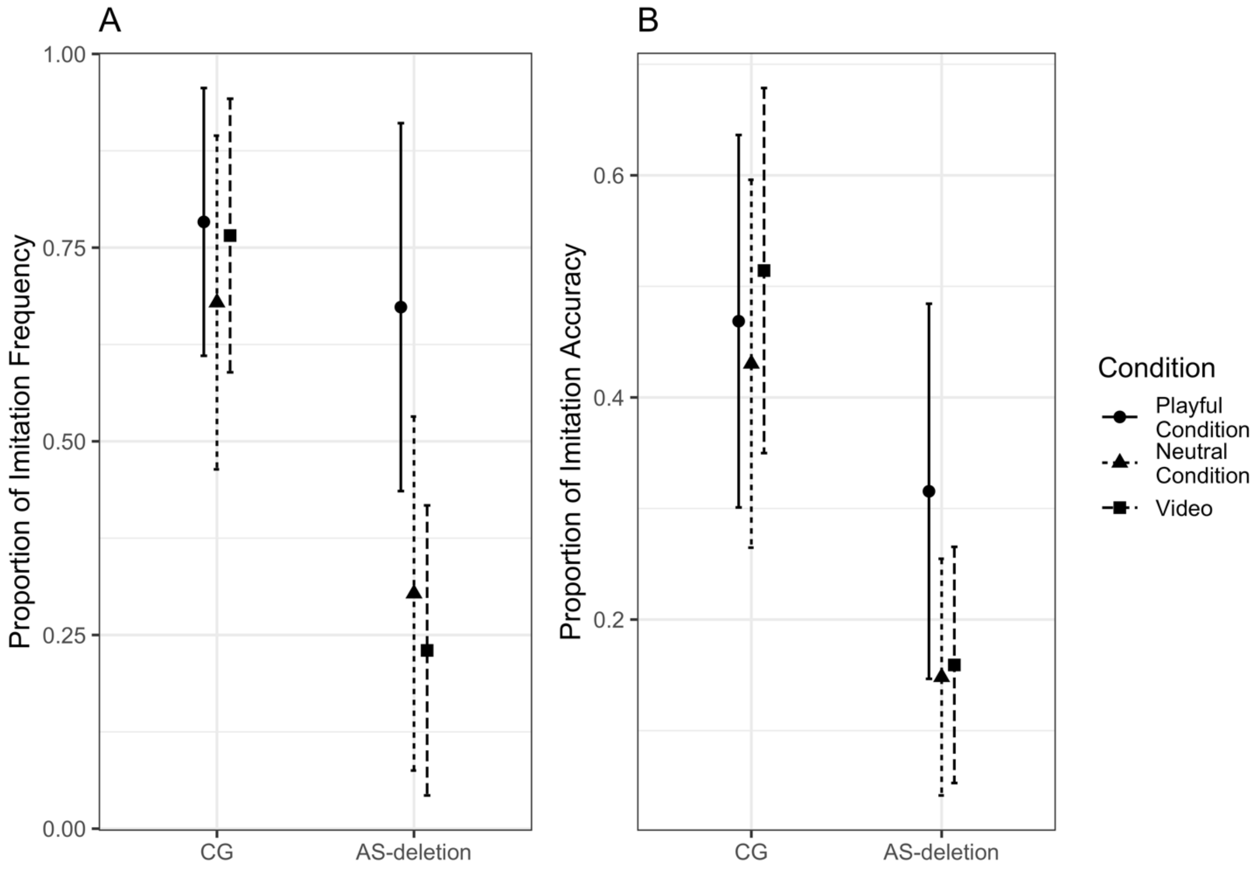

FIGURE 1S: Bar-plots of the proportion of imitation frequency (A) and accuracy (B) by group and condition estimated through a beta-binomial mixed effect model.

Study 2 – Imitation from a screen (2D)

In response to video-recorded demonstrations of actions, the frequency of imitation in the AS-deletion group was 31.25% (95% CI= 12.94-49.56), while accuracy was 21.35% (95% CI= 7.32-35.39). Like in the analyses that include the entire ASG, both imitation frequency and accuracy were significantly lower in the AS-deletion group than in the CG (p=0.001 across frequency and accuracy).

Different imitative behaviors in response to live versus video-recorded demonstrations

Similar to the analyses involving the entire ASG, across both imitation frequency and accuracy, the AS-deletion group had a better performance in response to live playful models, compared to video-recorded playful models (p<0.001, p=0.041 respectively). Figure 1S

Frequency and accuracy of imitation in association to Mental Age

When considering the effect of mental age on imitation frequency and accuracy we still found a positive effect in AS-deletion group but not statistically significant (p=0.178, p=0.297 respectively). When we considered the conditions separately we found a positive significant association between MA and imitation frequency in the Playful Condition in the AS-deletion group (p=0.038), similar to what we found when the entire ASG was considered. However, unlike in the original analyses, the association between the mental age of AS-deletion group and imitation accuracy did not reach statistical significance. Figure 2S, Table 1S-Table 2S.

Finally, similar to the results in the original analyses, when we compared the two groups in terms of the association between mental age and imitation accuracy we did not find any significant difference for both imitation frequency and accuracy and when considering the conditions separately. Figures and tables using only the deletion subgroup are reported below. Figure 2S, Table 1S-Table 2S.

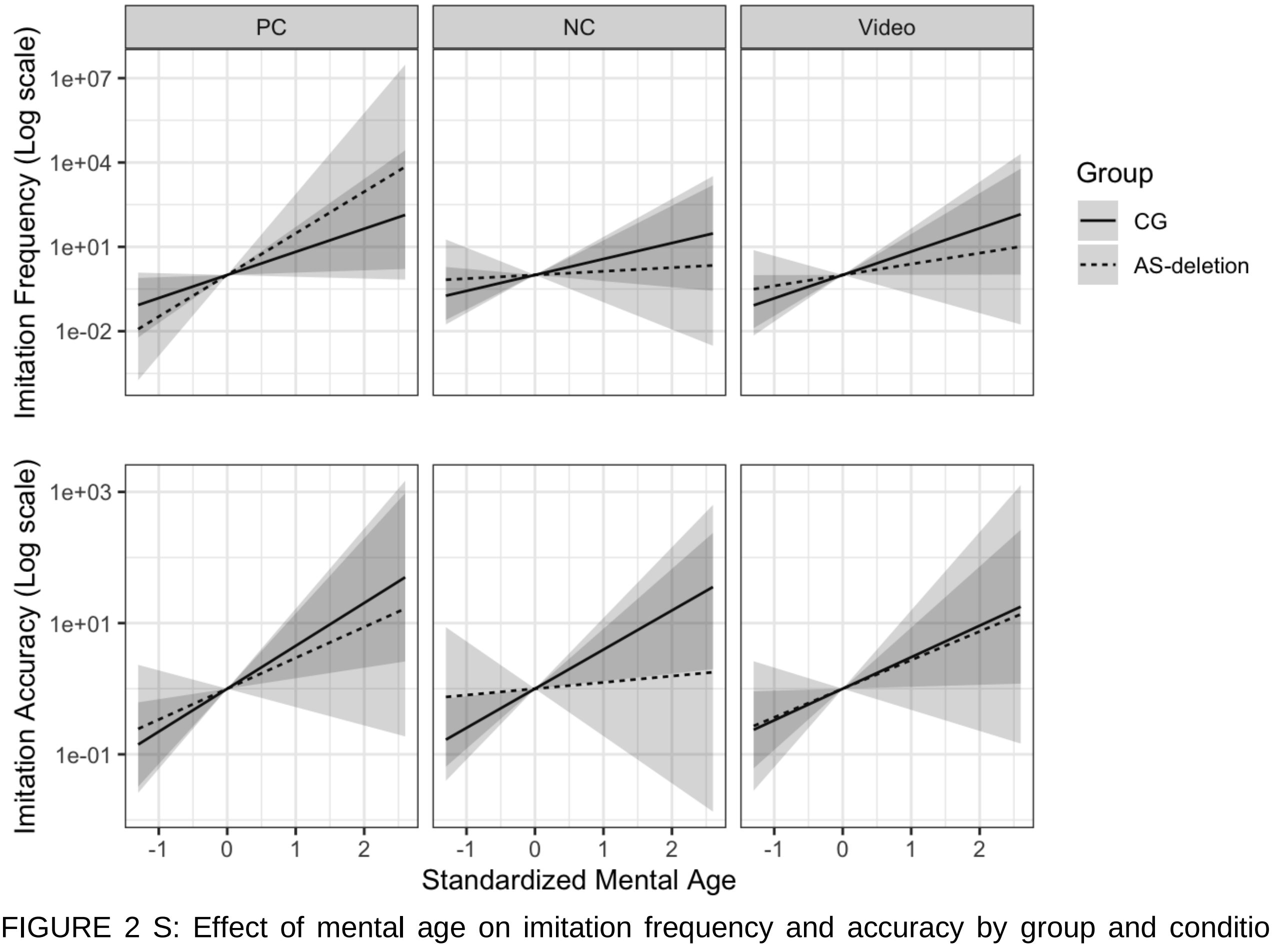

FIGURE 2 S: Effect of mental age on imitation frequency and accuracy by group and condition estimated through a beta-binomial mixed effect model. A log transformation is applied for the y axis.”

TABLE 1 S

	Mental Age CG<br>*Effect (CI); p-value*	***Mental Age AS-deletion group***<br>*Effect (CI); p-value*	***Mental Age AS-deletion-group –CG***<br>*Effect (CI); p-value*
Imitation Frequency Total Live	**1.00 (-0.14, 2.14)**<br>**0.083**	1.53 (-0.71, 3.78)<br>0.178	-0.17 (-2.89, 2.56)<br>0.902
Imitation Accuracy Total Live	**1.25 (0.43, 2.06)**<br>**0.004**	0.77 (-0.69, 2.23)<br>0.297	-0.56 (-2.27, 1.15)<br>0.518
Imitation Frequency Live neutral condition	1.26 (-0.45, 2.97)<br>0.146	0.30 (-2.23, 2.83)<br>0.814	-1.01 (-4.11, 2.10)<br>0.522
Imitation Accuracy Live neutral condition	**1.37 (0.29, 2.45)**<br>**0.014**	0.22 (-1.66, 2.10)<br>0.815	-1.15 (-3.34, 1.03)<br>0.298
Imitation Frequency Live playful condition	1.85 (-0.08, 3.79)<br>0.061	**3.41 (0.19, 6.63)**<br>**0.038**	1.52 (-2.24, 5.27)<br>0.425
Imitation Accuracy Live playful condition	**1.50 (0.39, 2.60)**<br>**0.009**	1.08 (-0.64, 2.81)<br>0.216	-0.42 (-2.49, 1.64)<br>0.685

Results of the effect of Mental Age in the AS-deletion group and in the Comparison Group (CG) on imitation frequency and accuracy in study 1 (3D) and the effect difference between the two groups. A beta-binomial regression was used for both Total scores and live playful, neutral and video-recorded playful conditions.

TABLE 2 S

	Mental Age CG<br>*Effect (CI); p-value*	***Mental Age AS-deletion group***<br>*Effect (CI); p-value*	***Mental Age AS-deletion group –CG***<br>*Effect (CI); p-value*
Imitation Frequency video recorded playful condition	1.86 (0.07, 3.65)<br>0.042	0.89 (-1.56, 3.35)<br>0.471	-1.02 (-4.12, 2.09)<br>0.517
Imitation Accuracy Video recorded playful condition	**1.10 (0.10, 2.11)**<br>**0.032**	1.01 (-0.74, 2.75)<br>0.256	-0.10 (-2.13, 1.93)<br>0.921

Results of the effect of Mental Age (MA) in the AS-deletion group and in the Comparison Group (CG) on imitation frequency and accuracy in study 2 (2D) and the effect difference between the two groups. A beta-binomial mixed effect model was applied when considering live playful, neutral and video-recorded playful conditions.
